# Clinical Performance of Infrazygomatic Crest Miniscrews Placed Without Surgical Guides: A Retrospective Study with 30-Month Follow-Up

**DOI:** 10.3390/bioengineering13080889

**Published:** 2026-07-31

**Authors:** Gianna Dipalma, Alessio Danilo Inchingolo, Maral Di Giulio Cesare, Sharon Di Serio, Marialuisa Longo, Francesco Inchingolo, Marco Severino, Ioana Roxana Bordea, Grazia Marinelli, Angelo Michele Inchingolo

**Affiliations:** 1 Interdisciplinary Department of Medicine, University of Bari “Aldo Moro”, 70124 Bari, Italy; gianna.dipalma@uniba.it (G.D.); alessiodanilo.inchingolo@uniba.it (A.D.I.); maral.digiuliocesare@uniba.it (M.D.G.C.);; 2 Department of Life Science, Health, and Health Professions, Link Campus University Italy Macerata, 00165 Rome, Italy; 3 Department of Medicine and Surgery, University of Perugia, Severi Square n.1, 06132 Perugia, Italy; 4 Department of Oral Rehabilitation, Faculty of Dentistry, Iuliu Hatiegan University of Medicine and Pharmacy, 400012 Cluj-Napoca, Romania; 5 Department of Biomedical, Surgical and Dental Sciences, Milan University, 20122 Milan, Italy

**Keywords:** orthodontic anchorage procedures, bone screws, maxilla, zygoma, malocclusion, retrospective studies

## Abstract

Background: Infrazygomatic crest (IZC) miniscrews provide reliable extra-alveolar skeletal anchorage for orthodontic tooth movement while minimizing dependence on patient compliance. Although guided insertion techniques have been proposed to improve placement accuracy, freehand insertion remains widely used because of its simplicity and clinical practicality. This retrospective study evaluated the clinical performance and survival of freehand-placed IZC miniscrews over a minimum follow-up period of 30 months. Materials and Methods: A retrospective observational study was conducted on 23 consecutive patients receiving a total of 30 IZC miniscrews between January 2022 and July 2024. All miniscrews were placed freehand by the same experienced orthodontist following individualized radiographic planning based on panoramic radiography and/or cone-beam computed tomography (CBCT), according to the patient’s anatomical requirements. Miniscrews were immediately loaded using light orthodontic forces (<100 g), with progressive force increases during subsequent appointments based on treatment objectives. Patients were reviewed at approximately 30–35-day intervals. Clinical records were analyzed to assess miniscrew survival, timing of failure, and procedure-related complications using descriptive statistical analysis. Results: Twenty-seven of the 30 miniscrews remained clinically stable throughout treatment, corresponding to an overall success rate of 90.0%. Three failures occurred during the early treatment phase, while no late failures were observed. Because of the limited number of failure events, potential contributing factors—including screw dimensions, mechanical interference, and deterioration of oral hygiene during treatment—were evaluated descriptively and should be interpreted with caution. No major surgical or radiographic complications were detected during follow-up. Conclusions: Within the limitations of this retrospective observational study, freehand placement of IZC miniscrews demonstrated favorable clinical performance when performed by an experienced operator following appropriate radiographic planning. However, the relatively small sample size, absence of a control group, and descriptive nature of the analysis preclude definitive conclusions regarding factors influencing miniscrew failure or the comparative effectiveness of freehand versus guided insertion. Larger prospective controlled studies are warranted to validate these preliminary findings. Clinical relevance Manual IZC miniscrew insertion represents a predictable, efficient, and cost-effective alternative to guided approaches in orthodontic practice.

## 1. Introduction

Temporary anchorage devices (TADs) have revolutionized orthodontic biomechanics by enabling skeletal anchorage independent of patient compliance [1].

Temporary anchorage devices (TADs) have significantly expanded the possibilities of orthodontic biomechanics by providing skeletal anchorage that is independent of patient compliance. Their introduction has allowed predictable management of complex tooth movements while reducing the need for patient-dependent extraoral appliances.

Among extra-alveolar sites, the infrazygomatic crest (IZC) has gained increasing attention due to its favorable anatomical characteristics, including increased cortical bone thickness and distance from dental roots, allowing placement of longer and more stable miniscrews [2].

Compared with conventional interradicular insertion sites, the infrazygomatic crest provides a larger bone volume and greater distance from adjacent dental roots, facilitating placement of longer miniscrews with potentially improved primary stability [3].

The IZC region has become increasingly popular in contemporary orthodontics because it allows the application of complex biomechanics without interfering with dental movement [4]. Compared with interradicular miniscrews, extra-alveolar anchorage systems provide greater flexibility for distalization mechanics, intrusion, and correction of asymmetries.

Consequently, IZC anchorage has become an established option for maxillary arch distalization, intrusion mechanics, correction of asymmetries, and treatment protocols requiring absolute anchorage without compromising the dental roots [5].

Furthermore, the increased cortical bone thickness typically observed in the infrazygomatic region may contribute to improved primary stability and reduced risk of root damage [6]. Nevertheless, the anatomical variability of the maxillary sinus and surrounding structures still represents a clinical challenge, particularly when freehand insertion techniques are used without digital guidance systems [7].

Despite these anatomical advantages, the proximity of the maxillary sinus, individual variations in bone morphology, and differences in cortical bone thickness require careful preoperative assessment and accurate surgical planning to minimize the risk of complications and optimize primary stability [8].

The literature reports success rates ranging from approximately 70% to 97%, depending on insertion protocols and patient-related variables [9].

Reported survival rates vary considerably among studies because of differences in patient selection, insertion protocols, loading strategies, operator experience, and duration of follow-up.

Recent advances in digital workflows have introduced CBCT-guided surgical templates to improve insertion accuracy and reduce complications [10]. However, clinical evidence suggests that satisfactory outcomes can also be achieved with conventional freehand techniques when performed by experienced clinicians with proper radiographic planning [11].

The increasing availability of cone-beam computed tomography (CBCT), intraoral scanning, and computer-assisted surgical guides has further improved insertion accuracy by enabling three-dimensional planning and more precise control of insertion angulation. Nevertheless, these digital approaches require additional equipment, increase treatment costs, and they may not always be necessary in routine clinical practice [12].

Several clinical studies have reported satisfactory outcomes with conventional freehand insertion when appropriate radiographic planning and adequate operator experience are available [13].

However, despite the widespread clinical use of freehand IZC miniscrew placement, evidence regarding its long-term clinical performance remains relatively limited, particularly in studies with follow-up periods extending beyond 2 years. Furthermore, few investigations have specifically evaluated clinical outcomes obtained without the use of surgical guides under routine clinical conditions [14].

Therefore, the aim of the present retrospective observational study was to evaluate the clinical performance of infrazygomatic crest miniscrews placed using a freehand technique by an experienced operator, focusing on survival, timing of failure, procedure-related complications, and long-term clinical stability during a minimum follow-up period of 30 months [15].

## 2. Materials and Methods

### 2.1. Study Design and Patient Selection

This retrospective observational study was conducted utilizing clinical and radiographic records from patients who underwent orthodontic treatment involving infrazygomatic crest (IZC) miniscrew insertion at the Interdisciplinary Department of Medicine, University of Bari “Aldo Moro”, between January 2021 and December 2023.

To minimize selection bias, a consecutive sampling approach was adopted: all consecutive patients who met the inclusion criteria within the specified timeframe were enrolled, resulting in a final sample of 23 patients and a total of 30 IZC miniscrews. The study was conducted in accordance with the Declaration of Helsinki, and informed consent was obtained from all participants or their legal guardians prior to treatment. This study was reported following the STROBE guidelines (Supplementary Materials File S1).

### 2.2. Sample Characteristics

Number of patients: 23
Age range: 15–40 years
Total miniscrews: 30
Miniscrew characteristics: Miniscrew diameter ranged from 1.6 to 2.3 mm, while length ranged from 8 to 15 mm.
Follow-up:
Mean follow-up period: 30 months
Cone-Beam CTCS 9600 (Carestream Dental LLC, Atlanta, GA, USA)
Panoramic radiograph (Carestream)
Manual screwdrive: PSM
Anesthetic used: Scandonest 2% with Adrenaline 1:100.00
Software used: CS 3D Imaging Software (Carestream Dental)

Patient demographics, miniscrew distribution, and failure analysis are summarized in Table 1.

### 2.3. Inclusion Criteria

- Patients requiring IZC skeletal anchorage;
- Complete clinical and radiographic records;
- Minimum follow-up ≥ 12 months;
- Age ≥ 14 years;
- Requirement for maxillary absolute anchorage (e.g., maxillary arch distalization, canine impaction traction, or anterior intrusion);
- Good general systemic health with no contraindications to minor oral surgery.

### 2.4. Exclusion Criteria

- History of systemic bone diseases or radiation therapy in the maxillofacial region;
- Active periodontal disease or poor oral hygiene at the baseline of the study (before treatment onset);
- Incomplete clinical or radiographic follow-up records.

### 2.5. Preoperative Assessment

Preoperative assessment was performed using CBCT and panoramic radiography to evaluate bone availability and anatomic structures. Preoperative diagnostic imaging was not identical for all patients and was strictly based on clinical indications. Digital panoramic radiographs were utilized for general screening and root-alignment assessment. In cases presenting challenging anatomy, high risk of root proximity, or suspected sinus floor cortical thinness, a Cone-Beam Computed Tomography (CBCT) scan was performed to accurately evaluate predictable bone volume and three-dimensional anatomic relations, thereby enhancing planning accuracy.

### 2.6. Surgical Protocol

All infrazygomatic crest miniscrews were placed freehand by the same orthodontist, who had previous clinical experience with more than 30 IZC miniscrew placement procedures before the beginning of the present study. The use of a single experienced operator minimized procedural variability and ensured consistency in surgical planning, insertion technique, and postoperative management.

Insertion parameters:

- Height: 11–17 mm above occlusal plane
- Angulation: 40–70°

Insertion was performed in the infrazygomatic crest region, typically between the first and second maxillary molars.

Following local infiltration anesthesia, the miniscrews were inserted into the infrazygomatic crest area. The insertion site was located approximately 11–17 mm above the maxillary occlusal plane, and the screws were driven with a manual driver at an angulation of 40–70° relative to the occlusal plane. These ranges represent targeted intraoperative clinical guidelines and were approximated clinically during the freehand procedure based on preoperative radiographic references. The miniscrews used in this study (Dual-Top, Jeil Medical Corporation, Seoul, Republic of Korea) consisted of titanium alloy (Ti-6Al-4V) with a diameter of 2.0 mm and lengths of 10, 12, or 14 mm, with the exception of one smaller screw (1.6 × 8 mm) used due to localized anatomical restrictions.

Loading Protocol:

All miniscrews were immediately loaded using light orthodontic forces (<100 g) to minimize excessive mechanical stress during the initial healing period. During subsequent follow-up appointments, orthodontic forces were progressively increased according to the planned biomechanics, generally exceeding 150 g once adequate clinical stability had been confirmed.

Follow up:

Patients were reviewed at regular intervals of approximately 30–35 days throughout active orthodontic treatment. At each visit, miniscrew stability was clinically assessed by evaluating mobility, peri-implant soft tissue conditions, patient discomfort, and the ability of the miniscrew to withstand the prescribed orthodontic force.

### 2.7. Outcome Measures

The primary outcome of the study was the clinical success rate of IZC miniscrews. The primary outcome of this study was the clinical success rate of the IZC miniscrews over the 30-month follow-up period. Clinical success was defined as the maintenance of miniscrew stability without pain, infection, or pathologic mobility, allowing for the successful completion of the planned orthodontic mechanics.

Secondary outcomes included the timing of failure, the influence of screw dimensions, and the occurrence of complications. The specific complications systematically monitored and recorded as present or absent at each appointment included: post-insertion persistent pain, soft tissue inflammation or infection (perimini-implantitis), soft tissue overgrowth covering the screw head, mechanical interference with the moving orthodontic appliance, and sinus-related pathology. Systematic clinical evaluations were used to screen for sinus symptoms; post-treatment radiographic follow-up (panoramic orlocalized intraoral radiographs) was routinely executed at the end of treatment or in case of suspected complications to definitively rule out root damage or sinus penetration.

### 2.8. Statistical Analysis

Descriptive statistics were used to summarize the data. Miniscrew survival was evaluated over the follow-up period, and failure timing (early versus late) was recorded. Due to the limited sample size, no inferential statistical analysis was performed.

## 3. Results

### 3.1. Patient and Miniscrew Characteristics

A total of 23 patients (14 females, 9 males; mean age 16.4±2.1 years) who received 30 IZC miniscrews were analyzed. The baseline patient and miniscrew-level data are summarized in Table 2. For clarity, data have been separated into patient-level and miniscrew-level characteristics.

### 3.2. Failure Pattern

Regarding the timing of failure, two miniscrews failed early, within the first 4 months of loading, while one failure occurred at Month 14 of treatment. All three failures were recorded on the left side. Given the limited number of failure events, this finding is reported as a descriptive observation only and was not subjected to statistical analysis.

### 3.3. Complications

The specific incidence of complications assessed during the 30-month follow-up is reported below, clarifying their presence or absence in the sample:

- Persistent Post-Insertion Pain: Absent. No patients reported pain lasting more than 48 h post-surgery.
- Sinus Pathology or Maxillary Sinusitis: Absent. No clinical symptoms of sinus pathology were reported, and post-treatment radiographic screening confirmed the absence of sinus floor alterations or mucosal thickening.
- Root Damage: Absent. Follow-up radiographs showed no structural root lesions or pericemental space alterations.
- Soft Tissue Overgrowth: Present in two cases (6.7%), managed successfully through localized hygiene enforcement and minor tissue trimming without removing the screw.
- Perimini-implantitis/Localized Inflammation: Present in one case (associated with early failure). Crucially, while poor oral hygiene was an exclusion criterion at baseline, this specific patient developed severe, persistent, neglected hygiene and plaque accumulation during the active treatment follow-up phase, leading to acute local inflammation and loss of primary stability.
- Mechanical Interference: Present in one case (associated with late failure), where direct contact occurred between the moving orthodontic archwire auxiliary and the miniscrew head during distalization mechanics, leading to secondary mobili

Secondary outcomes included failure timing and the occurrence of complications. Clinical success was defined as stability of the miniscrew throughout the orthodontic treatment period, whereas failure was defined as mobility or loss of the miniscrew, requiring removal.

### 3.4. Descriptive Factors Associated with Failures

Because the sample sizes among the size sub-groups are highly unequal and the total number of failures is extremely small (n=3), no inferential statistical analysis could be performed. The groups are highly unbalanced, and the failure of the single, small-dimension screw (1.6 × 8 mm) represents an isolated case. Therefore, these data must not be interpreted as evidence of a clear statistical association or a trend, but rather as descriptive observations to be treated with extreme caution.

The relationship between miniscrew dimensions and clinical outcomes is summarized in Table 3.

### 3.5. Figure Captions

**Figure 1:**
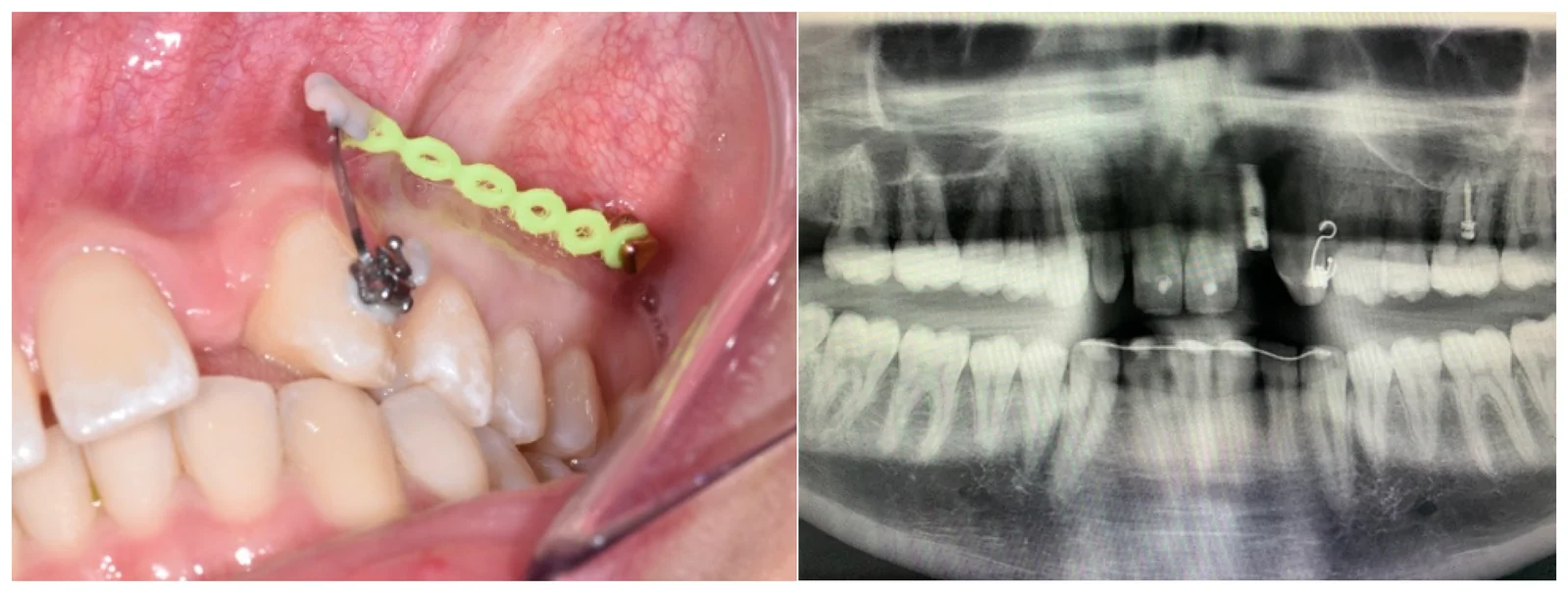
Case 1: Failure due to insufficient diameter and length of the screw (1.6 × 8 mm).

**Figure 2:**
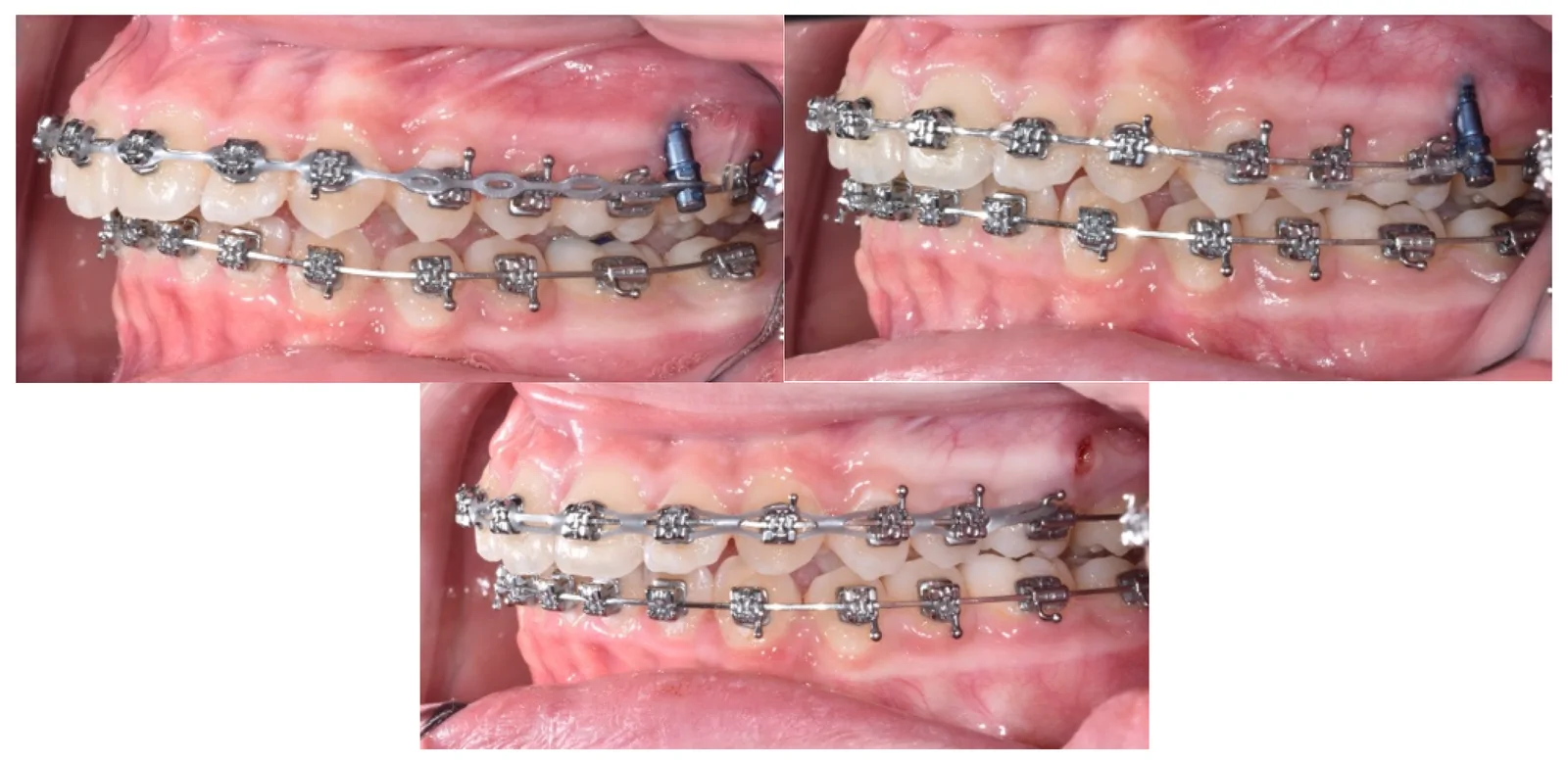
Case 2: Failure due to contact between the screw (2.3 × 13 mm) and the appliance during distalization.

**Figure 3:**
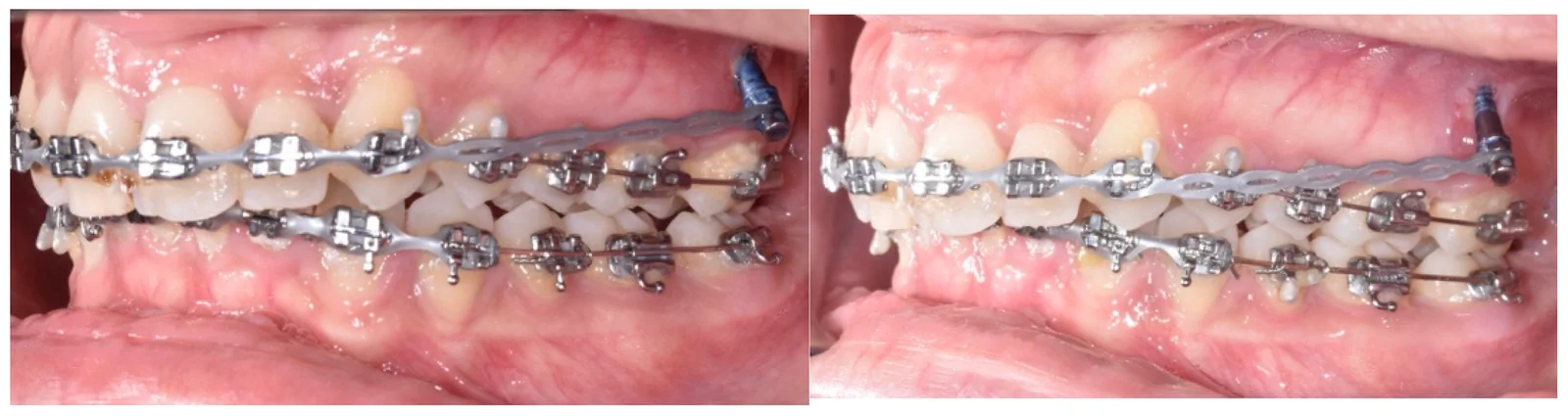
Case 3: Failure due to poor hygiene resulting in peri-implantitis of the screw (2.3 × 15 mm).

The following descriptive captions have been updated to guide the reader through the specific clinical and radiographic signs shown in each image: Figure 1: Preoperative radiographic planning. Cone-beam computed tomography (CBCT) cross-sectional view utilized to evaluate the specific bone thickness at the infrazygomatic crest area and to map the precise distance between the buccal cortical plate and the maxillary sinus floor prior to freehand insertion. Figure 2: Clinical view of freehand IZC miniscrew placement. Intraoperative view showing the horizontal insertion height relative to the mucogingival junction and the macroscopic clearance maintained between the miniscrew head and the active components of the orthodontic appliance. Figure 3: Radiographic sign of mechanical interference (Failure Case 2). Follow-up panoramic radiograph demonstrating direct structural contact between the distalizing orthodontic auxiliary wire and the body of the miniscrew, which generated cumulative microtrauma and led to progressive late loosening.

## 4. Discussion

### 4.1. Evaluation of Freehand IZC Miniscrew Insertion

The clinical efficacy of infrazygomatic crest (IZC) miniscrews as temporary anchorage devices (TADs) has revolutionized orthodontic biomechanics, particularly for maxillary arch distalization. Our findings demonstrated an overall clinical success rate of 90% over a 30-month follow-up period [16]. Within the limitations of this retrospective study, freehand IZC miniscrew placement showed favorable outcomes; however, this approach should not be generalized as superior or equivalent to guided protocols but rather framed as a viable alternative when performed with comprehensive preoperative radiographic planning and sufficient clinical experience [17,18,19].

### 4.2. Anatomical and Biomechanical Considerations

The IZC area presents distinct anatomical features, characterized by thick cortical bone supported by the zygomatic process of the maxilla [20]. Proper angulation during freehand insertion (40–70° relative to the occlusal plane) is crucial to maximize bone-to-implant contact while avoiding the maxillary sinus floor and adjacent molar roots [21,22]. While computer-guided templates inherently standardize these parameters, our data suggest that detailed preoperative assessment—using panoramic radiographs for baseline screening and CBCT for complex root or sinus anatomies—allows experienced clinicians to navigate these restrictions safely without routine template manufacturing [23].

### 4.3. Interpretation of Screw Dimensions

In our sample, 29 out of 30 miniscrews had a diameter of 2.0 mm, with only one smaller screw (1.6 × 8 mm) placed due to local anatomical limitations [24]. Although this single smaller screw failed, this finding must be interpreted with extreme caution due to the highly unequal distribution of the groups and the lack of a statistical sample size [25]. The failed small-dimension screw may tentatively suggest a possible role of screw size in primary stability, but this case represents an isolated descriptive event that cannot support a definitive clinical trend [26].

### 4.4. Analysis of Unilateral Failure Patterns

Interestingly, all three clinical failures in this cohort occurred on the left side. Because our total number of failures is small (n=3), any specific lateralization pattern lacks statistical power and cannot be correlated to a concrete risk factor [27]. While we hypothesize that operator positioning and subtle direct visibility differences during right-handed freehand insertion might play a role, this explanation remains strictly speculative and must be treated purely as an unproven hypothesis [28].

### 4.5. Biological Stability vs. Mechanical Retention

Unlike standard osseointegrated dental implants, orthodontic miniscrews rely almost entirely on primary mechanical retention rather than macro-biological integration. Consequently, their stability is highly susceptible to external perturbations. We observed two distinct failure mechanisms in our sample: biological loss of stability and mechanical interference [29].

- Biological Failure (Case 1): Severe perimini-implantitis occurred due to localized plaque accumulation. It is important to clarify that this patient met the strict inclusion criteria of good baseline oral hygiene before treatment. However, severe poor hygiene and neglect developed downstream during the active orthodontic follow-up phase, generating acute soft tissue inflammation that rapidly compromised mechanical retention [30].
- Mechanical Failure (Case 2): Late failure occurred at month 14 due to direct contact between the moving orthodontic archwire auxiliary and the miniscrew body during distalization. This represents a preventable clinical error, highlighting that continuous monitoring of the clearance between active orthodontic components and the TAD head is essential during monthly reviews to avoid cumulative microtrauma [31].

### 4.6. Safety Profile and Complications

The clinical safety profile of the freehand technique in this study was favorable, as no major intraoperative or postoperative complications were recorded. Despite the lack of physical guidance, systematic clinical screening and post-treatment radiographic validation confirmed the complete absence of long-term root damage or maxillary sinus pathology [32,33]. This safe profile is highly dependent on the operator’s clinical touch and adherence to established safe vertical insertion windows. Soft tissue overgrowth (n=2) was easily managed conservatively, confirming that minor mucosal complications do not necessarily endanger the overall anchorage integrity [34].

### 4.7. Clinical Recommendations for Freehand Placement

Based on our retrospective data, freehand IZC placement can achieve high success rates if a structured clinical protocol is followed: rigorous patient selection, careful individual mapping of the IZC anatomy via appropriate imaging, and strict adherence to immediate loading forces not exceeding 150–200 g. Furthermore, monthly physical stability checks and immediate intervention upon signs of auxiliary wire interference or slipping oral hygiene are critical to prevent early or late failure cascades [35,36].

### 4.8. Limitations of the Study

Several limitations must be explicitly considered when interpreting our results. First, this study is retrospective and descriptive, based on a limited sample size of 23 patients and 30 miniscrews, which precluded advanced inferential statistical analysis to isolate independent risk factors. Second, the study completely lacks a direct control group undergoing guided template insertion, making absolute comparative claims impossible. Third, a potential selection bias cannot be entirely ruled out despite consecutive sampling. Moreover, sample size was determined by convenience sampling based on the consecutive patients meeting the inclusion criteria during the specified timeframe. Finally, because all procedures were carried out by a single operator heavily experienced in IZC placements, the high success rates reported here are highly operator-dependent and may not be easily replicable by clinicians still on the early phases of their learning curve [37,38,39,40].

## 5. Conclusions

Within the limitations of this retrospective, a descriptive study based on a limited sample size, freehand IZC miniscrew placement showed favorable outcomes when performed with appropriate radiographic planning and clinical experience. A success rate of 90% was observed over a 30-month follow-up period, with no late failures. Although descriptive observations tentatively suggest a potential role of screw dimensions, mechanical interference, and oral hygiene maintenance in early failures, the extremely small number of failure events (n=3) precludes any definitive conclusions regarding these contributing factors. Furthermore, the absence of a guided-control group prevents direct comparisons between freehand and guided insertion techniques. Therefore, while freehand IZC placement represents a viable and clinically effective option for skeletal anchorage in experienced hands, these findings should not be broadly generalized to all clinical settings. Larger prospective, controlled studies are necessary to validate these outcomes and accurately isolate specific risk factors.

## Figures and Tables

**Table 1:** Demographic characteristics, miniscrew distribution and failure analysis.

Characteristic	*n* (%)
**Patients**	23 (100)
**Sex**	
Female	14 (60.9)
Male	9 (39.1)
Age Range (years)	15–40
**Miniscrews placement**	30 (100)
Bilateral	20 (66.7)
Unilateral	10 (33.3)
**Failures**	3 (10.0)
**Failure Analysis**	
Recued screw dimensions (1.6 × 8 mm)	1 (3.3)
Mechanical interference	1 (3.3)
Poor oral hygiene	1 (3.3)

**Table 2:** Baseline characteristics of the study sample.

Level	Parameter	Specification/Value	Distribution (n)
Patient-Level Data (n = 23)	Mean Age	16.4 ± 2.1 years	—
	Gender	Female	14
	Gender	Male	9
Miniscrew-Level Data (n = 30)	InsertionSide	Right	15
	InsertionSide	Left	15
	Screw Diameter	2.0 mm	29
	Screw Diameter	1.6 mm	1
	Screw Length	8 mm	1
	Screw Length	10 mm	4
	Screw Length	12 mm	18
	Screw Length	14 mm	7

**Table 3:** Miniscrew dimensions and associated clinical outcomes.

Miniscrew Characteristic	n	Successful	Failed
Length (mm)			
8	1	0	1
13	20	19	1
15	9	8	1
Diameter (mm)			
1.6	1	0	1
2.3	29	27	2
Total	30	27	3

## Data Availability

The data presented in this study are available from the corresponding author upon reasonable request. The data are not publicly available due to privacy and ethical restrictions related to patient confidentiality.

## Supplementary Materials

The following supporting information can be downloaded at: https://www.mdpi.com/article/10.3390/bioengineering13080889/s1, File S1: STROBE Statement—Checklist of items that should be included in reports of cohort studies.
